# Twin pregnancy complicated with congenital Hemivertebra: report of two cases and literature review

**DOI:** 10.1186/s12884-020-03177-3

**Published:** 2020-08-20

**Authors:** Tingting Xu, Xiaodong Wang, Haiyan Yu, Fumin Zhao

**Affiliations:** 1 Department of Obstetrics and Gynecology, West China Second University Hospital, Sichuan University, Chengdu, China; 2 Key Laboratory of Birth Defects and Related Diseases of Women and Children (Sichuan University), Ministry of Education, Chengdu, China; 3 Department of Radiology, West China Second University Hospital, Sichuan University, Chengdu, China

**Keywords:** Fetal hemivertebrae, Dichorionic diamniotic twin pregnancy, Magnetic resonance imaging

## Abstract

**Background:**

Hemivertebra deformity, involving one or multiple vertebral bodies, is one of the important causes of congenital scoliosis. Congenital fetal hemivertebrae could be diagnosed by ultrasonography and confirmed by fetal magnetic resonance imaging during pregnancy. However, reports of hemivertebrae in twins during the perinatal period are very rare.

**Case presentation:**

We report two cases of congenital fetal hemivertebrae, each affecting one fetus in a dichorionic diamniotic (DCDA) twin pregnancy. We have also conducted a literature review of its prenatal screening, diagnosis, management, and outcomes. These two cases of congenital fetal hemivertebrae in one fetus of a DCDA twin were both initially found by ultrasonography and confirmed by fetal magnetic resonance imaging (MRI). One couple chose selective termination of the hemivertebrae fetus after they were extensively counseled by the multidisciplinary team regarding the treatment and prognosis of the hemivertebrae twin, and a healthy baby weighing 2320 g was delivered at the 37^+ 1^ gestational week. The other couple decided to continue the twin pregnancy and gave birth to two living newborns weighing 2580 g and 2060 g at 37^+ 1^ gestational weeks. These three babies were all in good health during follow-up.

**Conclusions:**

Based on our center’s experience, comprehensive ultrasonography is necessary for early prenatal diagnosis of this condition. In addition, fetal MRI will confirm the diagnosis of hemivertebrae and provide parents with helpful information for their decision about the fate of the affected fetus.

## Background

Hemivertebra is a rare congenital vertebral abnormality that can lead to deformation of the spine, such as scoliosis, lordosis, or kyphosis [1], and it is usually located in the thoracic and lumbar regions. The birth rate of hemivertebrae is 0.05–0.10% [2], occurring more commonly in females. The sex ratios (male/female) for multiple vertebral anomalies and solitary vertebral anomalies are respectively 0.31 and 0.68 [3].

The majority of published papers about hemivertebrae involve surviving newborns after birth, and few papers have focused on cases of congenital fetal hemivertebrae diagnosed during prenatal care. Most fetal hemivertebrae cases involve a singleton pregnancy, and cases of only one twin with hemivertebrae are very rare.

Based on the published data of fetal hemivertebra in singletons we can see that fetal hemivertebra can be divided into isolated cases and coexisting anomalies. Most fetuses with prenatally diagnosed hemivertebrae in singletons usually have coexisting anomalies, which could affect the prognosis of hemivertebra with high rates of cesarean delivery, growth restriction, and fetal/neonatal loss. Besides, nonisolated hemivertebrae of neonates usually born before term with higher mortality rates [4, 5]. The prognosis of fetal hemivertebra is related to the type, site, the number of the affected vertebra, and the associated anomalies. Isolated fetal hemivertebrae is considered to carry a good prognosis [6]. The presence of associated anomalies reduces the survival to 50%, and when accompanied by significant oligohydramnios, the mortality is 100% [6].

Here, we reported two cases of one fetus with congenital hemivertebrae in dichorionic diamniotic (DCDA) twin pregnancies at West China Second University Hospital, a tertiary referral center in west China, between January 2018 to March 2020. Additionally, we conducted a literature review about the perinatal management and postnatal outcomes of twin pregnancies compared with one fetus with prenatally diagnosed hemivertebrae.

## Case presentation

### Case 1

A 32-year-old woman, gravida 2, para 0, conceived dichorionic diamniotic (DCDA) twins by ovulation-induction therapy. The couple was not consanguineous and had no reported history of medication, substance abuse, or a family history of congenital anomalies. The patient’s serology was negative for HIV, VDRL, and HBsAg.

**Fig. 1:**
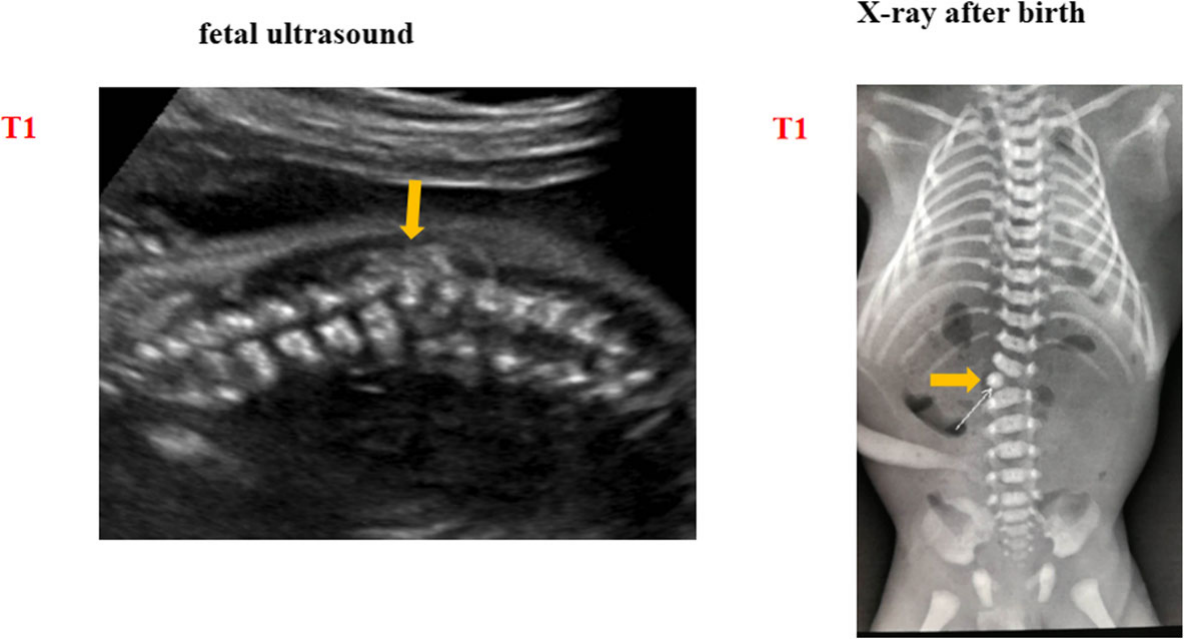
Imaging manifestations of the DCDA twins in case one. Panel 1 shows the fetal ultrasound of the congenital hemivertebrae twin (T1), and panel 2 shows the X-ray after the birth of T1. The **yellow arrow** indicates the defect of fetal hemivertebrae. T1 is short for the congenital hemivertebrae twin in case one

Prenatal ultrasonography identified the twin pregnancy with one fetus displaying hemivertebrae at 25^+^ weeks of gestation. Given this condition, the patient was transferred to our department. The ultrasonography in our hospital revealed the hemivertebrae in the lumbar (L) 2–3 vertebral bodies in one twin (Fig. 1 panel 1), while the other twin was healthy. Prenatal diagnosis of the fetus with hemivertebrae was subsequently confirmed by fetal magnetic resonance imaging (MRI), which was shown in Additional file 1, besides, the fetal MRI of these twin was shown in Additional file 2. The spine in the thoracolumbar junction area was slightly affected by scoliosis and the vertebral body morphological structures of L1 and L2 were abnormal with no other structural abnormalities, including musculoskeletal, genitourinary, cardiac, and so on.

The chromosomal microarray analysis of the amniotic fluid sample from both twins was normal. In addition, amniotic fluid samples from the two fetuses identified them as nonidentical twins. After the couple was extensively counseled by a multidisciplinary team regarding the treatment and prognosis of the hemivertebrae twin, the parents chose selective termination of the hemivertebrae fetus. Thus, legal termination of the affected fetus was performed by ultrasound-guided intrathoracic injection of KCl during the pregnancy based on local laws and religious beliefs.

At 37^+ 1^ gestational week, a cesarean section was performed due to a breech presentation in labor. A healthy female baby weighing 2320 g was delivered with Apgar scores of 10 and 10 at 1 and 5 min, respectively. The dead co-twin was a male baby weighing 1655 g. The parents agreed to postmortem radiological examination but refused an autopsy of the dead co-twin. The normal twin was healthy with no abnormality. The radiological examination confirmed the dead co-twin had hemivertebrae (Fig. 1 panel 2), while the healthy baby had a normal spine. The baby was in good health for more than 2 years of follow-up.

### Case 2

**Fig. 2:**
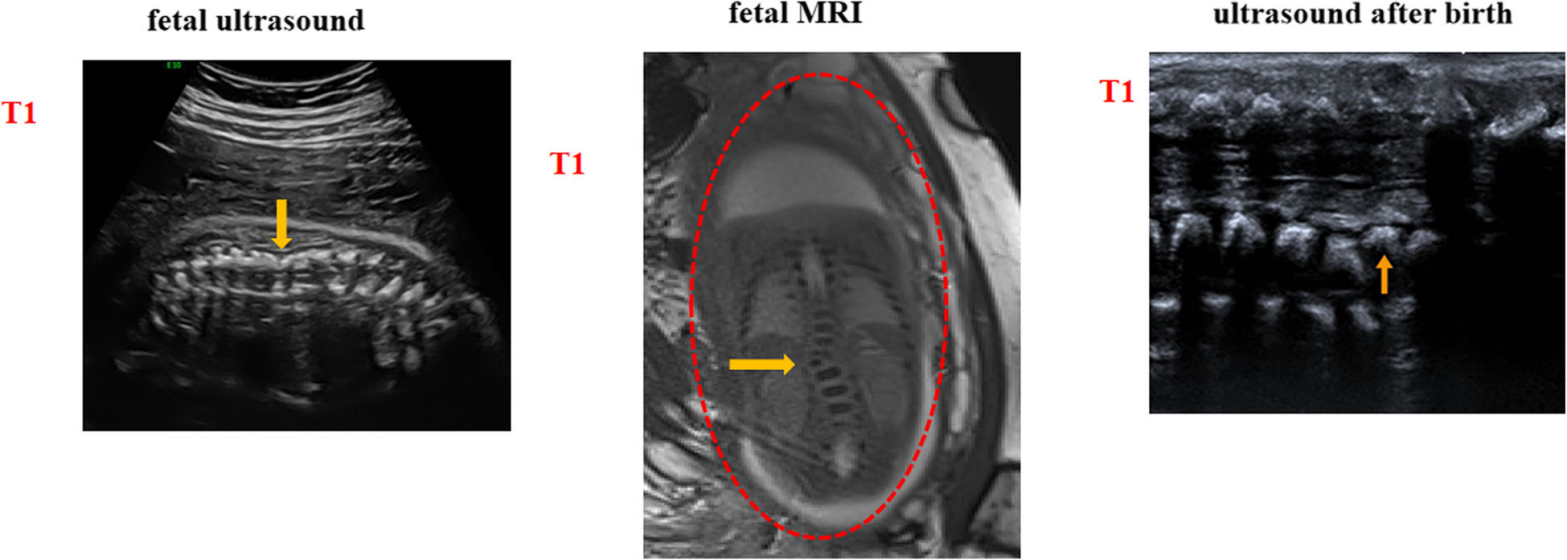
Imaging features of the DCDA twins in case two. Panel 1 shows the fetal ultrasound of the congenital hemivertebrae twin (T1), panel 2 shows the fetal MRI of T1, and panel 3 shows the ultrasound after the birth of T1. The yellow arrow indicates the defect of fetal hemivertebrae, and the dotted red line marks the area indicating the gravid uterus in the fetal MRI. T1 is short for the congenital hemivertebrae twin in case two

A 32-year-old pregnant woman, gravida 3, para 0, underwent in vitro fertilization and embryo transfer (IVF-ET), and 2 embryos were transferred to the uterus. After the embryo transfer, ultrasonography revealed a DCDA twin pregnancy. The patient’s serology was negative for HIV, VDRL, and HBsAg. The couple had no reported history of medication, substance abuse, or a family history of congenital anomalies. The regular prenatal ultrasonography at 25^+ 3^ gestational weeks identified that the T12 vertebral body of the spine was slightly abnormal in one twin (Fig. 2 panel 1) without any other structural abnormalities including musculoskeletal, genitourinary, cardiac, etc., while the co-twin was normal. Fetal MRI of twin was performed (Additional file 3)  and the diagnosis of fetal hemivertebrae was made for this affected fetus (Fig. 2 panel 2). The co-twin was normal. The chromosomal microarray analysis of the amniotic fluid sample in both twins was normal. The couple decided to continue the pregnancy after extensive counseling by the multidisciplinary team.

A cesarean section was conducted at 37^+ 1^ gestational weeks for a fetal breech presentation, and two living male babies were delivered. The birth weights of these two newborns were 2580 g and 2060 g with Apgar scores of 10 and 10 at 1 and 5 min, respectively. We explained to the parents the advantages and disadvantages of ultrasound (US), X-ray, and MRI examinations, and the parents were worried about the radiation of X-rays and the expense of MRI. Finally, they chose postnatal documentation of the hemivertebrae in the second twin by the postnatal US. We found the T12 level hemivertebrae (Fig. 2 panel 3) and no other structural abnormalities. The twins were in good health for more than 1 year of follow-up. The baby with hemivertebrae was assessed for associated skeletal, cardiac, renal, and gastrointestinal anomalies after birth and showed no progression of scoliosis. He is being followed by the Child Healthcare Department and Orthopedics Department.

## Discussion and conclusions

Hemivertebrae may be isolated or found in association with coexisting multiple anomalies [2, 7]. The exact etiology of hemivertebrae is unclear [7, 8], involving both genetic and environmental factors [9]. The incidence of karyotypic abnormalities in fetuses with isolated hemivertebrae is low [2, 8, 10]. In our study, chromosomal microarray analysis of the amniotic fluid sample from both twins of the included cases was conducted and no abnormal results were found.

Hemivertebrae is also a component of several genetic syndromes, including Jarcho-Levin syndrome (autosomal recessive; fused vertebrae, scoliosis, abnormal rib alignment), Klippel-Fiel syndrome (autosomal recessive or dominant; the fusion of the cervical vertebrae), VACTERLsyndrome (sporadic; vertebral and ventricular septal defects, anal atresia, tracheoesophageal fistula, renal anomalies, radial dysplasia, and single umbilical artery), and the OEIS complex (sporadic; omphalocele, exstrophy of the cloaca, imperforate anus, and spinal defects) [1, 3, 4, 7, 10, 11]. Therefore, it is very important to be specific about its identification and its correlations with other complications. Attention should be given to the clinical diagnosis and treatment of hemivertebrae. What’s more, the high risk of associated anomalies needs to be correctly disclosed and reported to the parents prenatally at the diagnosis of hemivertebra.

Routine prenatal ultrasonographic screening is the primary method used to identify fetal hemivertebrae; however, the diagnosis of hemivertebrae should be confirmed by fetal MRI and then post-birth or post mortem by X-ray examinations. Additional structural abnormalities, mainly musculoskeletal, genitourinary, and cardiac, are found in more than 70% of cases [2, 5, 7, 12]. Therefore, assessments for associated skeletal, cardiac, renal, and gastrointestinal anomalies should be performed once hemivertebrae is recognized [13].

In addition, fetal MRI is very useful in evaluating the entire spine. It can detect some abnormalities, especially central nervous system abnormalities, and add some additional useful information in complicated cases, for which the information is insufficient by ultrasonography [4, 11]. The fetal MRI results allow the fetal condition and prognosis to be evaluated in detail by a multidisciplinary team and further suggestions and advice can be given to the couple. In our study, fetal MRI of these two included cases was conducted to confirm the diagnosis of fetal hemivertebrae.

To the best of our knowledge, the globally published papers in English related to twin pregnancy with the hemivertebrae number only 4 so far [9, 14–16]. It has been found that in twin pregnancy, even in identical twins, if one fetus has congenital scoliosis, the other one can be normal [9]. However, there is no consensus on the perinatal management of twin pregnancies with hemivertebrae.

Sturm P. F., et al. [9] reported a case of monozygotic twins who both suffered from hemivertebrae with no other deformity. These twins were first evaluated for their spinal anomalies at age 11 months; however, both twins showed minimal progression of their spinal curves within two years of follow-up. Benacerraf B. R., et al. [14] reported two cases of twin pregnancies affected by hemivertebrae. One case delivered one hemivertebrae newborn and one healthy newborn. However, the other case was a blighted twin and only a single fetus with hemivertebrae was alive. Weisz B, et al. [5] reported two cases of DCDA twin pregnancies with one hemivertebrae fetus and one healthy fetus. Among these two cases, one case chose selective termination, and the other case delivered two living newborns without any neurological deficits within a 24 month follow up. Kaspiris A, et al. [16] reported a case where both twins had hemivertebrae, with congenital scoliosis, moderate mental retardation, and dyslalia. Detailed information of these cases is shown in Table 1.

**Table 1:** The character of the included study

Study ID	Maternal age (years)	Gravida	Para	Spontaneously conceived	Type of twin	Diagnose time	Gestational age (weeks)	Delivery method	Maternal complication	Pregnancy outcomes		affected hemivertebra	Side	Associated congenital abnormalities	karyotype
Sturm, P. F	NS	NS	NS	NS	monozygotic	11 months after delivery	35	VD	NO	Twin 1	Hemivertebra, alive	T7, T8	right	No	NS
										Twin 2	Hemivertebra, alive	T5, T6	left	No	NS
Benacerraf, B. R.	29	1	0	NS	NS	26 weeks	36	CS	PROM	Twin 1	Hemivertebra, alive	the upper lumbar vertebral bodies	NS	NS	NS
										Twin 2	Normal and alive				
Benacerraf, B. R.	23	2	1	NS	NS	17 weeks	37	CS	PROM	Twin 1	Hemivertebra, alive	T9	NS	NS	NS
										Twin 2	Died at 17 weeks				
Weisz, B.	37	2	1	ART	DCDA	15 weeks	NS	NS	NS	Twin 1	hemivertebra, selective termination	L3, L4	NS	single umbilical artery	(−)
										Twin 2	Normal and alive				
Weisz, B.	30	1	0	Yes	DCDA	14 weeks	NS	NS	NS	Twin 1	Alive, no neuro deficit	L2	NS	NO	(−)
										Twin 2	Normal and alive				
Kaspiris A	NS	NS	NS	NS	monozygotic	11 years old after birth	NS	NS	NS	Twin 1	incarcerated hemivertebrae, alive	Th6-Th10, Th 10-L2;	Left	a left thoracic scoliosis with an upper curve from Th6–10 and a lower curve Th10-L2 with a Cobb angle of 34°	NS
										Twin 2	incarcerated hemivertebrae, alive	Th9-L1	Left	a left thoraco lumbar scoliosis with a curve from Th9-L1 and a Cobb angle of 10°	NS
**Our cases**															
No. 1	32	2	0	ART	DCDA	25+ weeks	37 + 1	CS	No	Twin 1	hemivertebra, selective termination	L1-L2	right	No	(−)
										Twin 2	Normal and alive				
No. 2	32	3	0	ART	DCDA	25 + 3 weeks	37 + 1	CS	GDM	Twin 1	Hemivertebra, alive	Th11	left	No	(−)
										Twin 2	Normal and alive				

# *NS* Not specified, *Th* Thoracic, *L* Lumbar, *PROM* Premature rupture of membranes, *DCDA* Dichorionic diamniotic, *MCDA* Monochorionic diamniotic, *MCMA* Monochorionic monoamniotic, *CS* Cesarean section, *VD* Vaginal delivery, *ART* Assisted reproductive technology, *GDM* Gestational diabetes mellitus

From the above-published results and our center’s experience, we can see that all of these hemivertebrae cases were alive after birth except when the parents chose selective termination, and some had deformation of the spine; however, isolated hemivertebrae has a good prognosis. Hence, given the above available evidence, there is no different behavior of hemivertebra in twins as compared to singletons.

If only one vertebra is affected and no other fetal malformations are detected, conservative management of the pregnancy could be offered. Termination of pregnancy should only be offered if multiple vertebrae are malformed, or if anomalies in additional organ systems are seen [13]. The treatment and prognosis of hemivertebrae depend on the timing of its diagnosis, the location and number of the affected vertebra, and the associated anomalies [2, 8].

Fetuses with hemivertebrae have high rates of cesarean section, growth restriction, low birth weight, and/or preterm delivery [2]. In our study, these two cases were both delivered by cesarean section due to breech presentation of a twin pregnancy.

In conclusion, comprehensive ultrasonographic screening of the fetus allows an early prenatal diagnosis of hemivertebrae. The prognosis of isolated hemivertebrae is good and expectant management of the pregnancy can be adopted if no other major fetal malformations are detected. The prenatal ultrasound and MRI diagnosis of fetal hemivertebrae require careful and meticulous examinations, and this can provide parents with helpful information about their decision regarding the fate of the pregnancy. When the parents opt to continue with the pregnancy, a multidisciplinary team involving geneticists, obstetricians, pediatricians, neurosurgeons, spinal surgeons, physiotherapists, and psychologists is necessary. In addition, chromosomal analysis can be offered. Once identified, a careful neonatal assessment for associated cardiac and genitourinary anomalies needs to be performed. If there are no other complicating factors, standard management of labor and delivery is recommended.

## Supplementary information

**Additional file 1.** Fetal MRI of afftected fetus in case one.

**Additional file 2.** Fetal MRI of twin in case one.

**Additional file 3.** Fetal MRI of twin in case two.

## Data Availability

Not applicable for this case series.

## Abbreviations

DCDA: Dichorionic diamniotic
MRI: Magnetic resonance imaging
HIV: Human immunodeficiency virus
VDRL: Venereal disease research laboratory
HBsAg: Hepatitis B surface antigen
IVF-ET: In vitro fertilization and embryo transfer
