# Supplementary figures and images for: Twin pregnancy complicated with congenital Hemivertebra: report of two cases and literature review
Source: BMC Pregnancy Childbirth. 2020 Aug 20;20:479. doi: 10.1186/s12884-020-03177-3 (PMC7441669; doi:10.1186/s12884-020-03177-3)

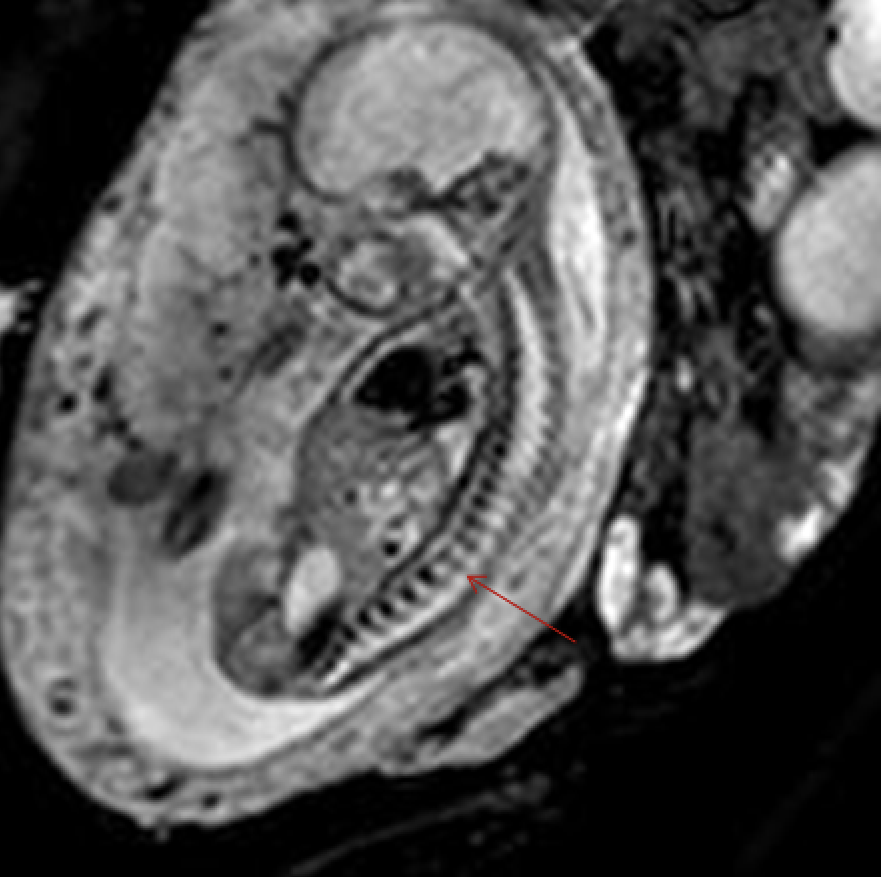

Supplement: Supplementary file 1 — Additional file 1. Fetal MRI of afftected fetus in case one. [file 12884_2020_3177_MOESM1_ESM.png]

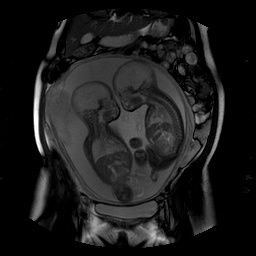

Supplement: Supplementary file 2 — Additional file 2. Fetal MRI of twin in case one. [file 12884_2020_3177_MOESM2_ESM.tif]

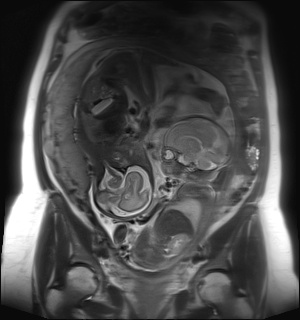

Supplement: Supplementary file 3 — Additional file 3. Fetal MRI of twin in case two. [file 12884_2020_3177_MOESM3_ESM.tif]
